# Supplementary material for: Tead1a Initiates Transcriptional Priming Through the TEAD1a/YAP‐Notch1‐Spi1/Cebpα Axis to Promote Neutrophil Fate
Source: Adv Sci (Weinh). 2025 Aug 28;12(41):e05441. doi: 10.1002/advs.202505441 (PMC12591127; doi:10.1002/advs.202505441)
Supplement: Supplementary file 4 — Supplementary Dataset [file ADVS-12-e05441-s004.zip › 3-1.zip!/3-1/02-1.cluster/cluster1/Enrich/ego_MF.pdf]

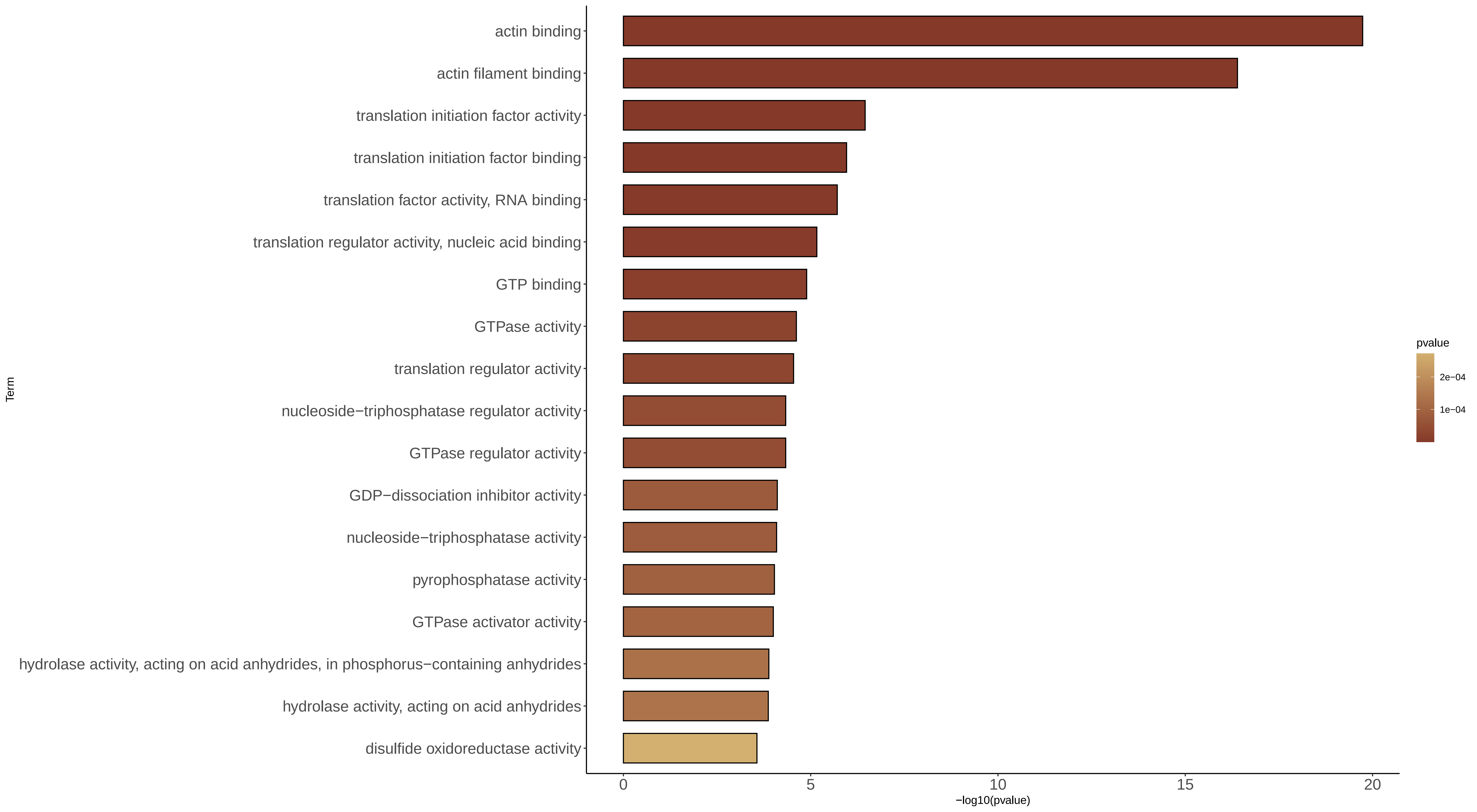
actin binding
actin filament binding
translation initiation factor activity
translation initiation factor binding
translation factor activity, RNA binding
translation regulator activity, nucleic acid binding
GTP binding
GTPase activity
translation regulator activity
nucleoside−triphosphatase regulator activity
GTPase regulator activity
GDP−dissociation inhibitor activity
nucleoside−triphosphatase activity
pyrophosphatase activity
GTPase activator activity
hydrolase activity, acting on acid anhydrides, in phosphorus−containing anhydrides
hydrolase activity, acting on acid anhydrides
disulfide oxidoreductase activity
Term
0
5
10
15
20
−log10(pvalue)
pvalue
2e−04
1e−04
